# PET imaging of FAAH in chronic Cannabis users: longitudinal assessment during short-term abstinence

**DOI:** 10.1038/s41386-026-02438-7

**Published:** 2026-05-09

**Authors:** Claudia Poluga, Shahtaj S. Dheda, Nadia Boachie, Tina McCluskey, Lucas Narciso, Maia Zilberman, Cassie Côté, Marilyn A. Huestis, Rachel F. Tyndale, Stefan Kloiber, Nathan J. Kolla, Pablo M. Rusjan, Bernard Le Foll, Jerry J. Warsh, Tony P. George, Isabelle Boileau

**Affiliations:** 1 Brain Health Imaging Centre and Campbell Family Mental Health Research Institute, Centre for Addiction and Mental Health, Toronto, ON Canada; 2 Department of Pharmacology and Toxicology, University of Toronto, Toronto, ON Canada; 3 Institute of Medical Science, Temerty Faculty of Medicine, Toronto, ON Canada; 4 Department of Psychiatry, Temerty Faculty of Medicine, University of Toronto, Toronto, ON Canada; 5 Institute for Emerging Health Professions, Thomas Jefferson University, Philadelphia, PA USA; 6 Forensic Behavioural Science, Swinburne University of Technology, Melbourne, VIC Australia; 7 Department of Psychiatry, McGill University, Montreal, QC Canada; 8 Douglas Research Centre (Human Neuroscience Division), Montreal, QC Canada; 9 Institute for Mental Health Policy Research, Centre for Addiction and Mental Health, Toronto, ON Canada; 10 Waypoint Centre for Mental Health Care, Penetanguishene, ON Canada

**Keywords:** Addiction, Risk factors, Addiction

## Abstract

Cannabis withdrawal in cannabis use disorder (CUD) increase the risk of relapse and lacks effective treatments. The endocannabinoid enzyme fatty acid amide hydrolase (FAAH) may influence cannabis use and withdrawal, but the relationship between FAAH levels and withdrawal symptoms remains unclear. This study aims to investigate changes in FAAH levels during short-term abstinence from cannabis and their relationship with withdrawal symptoms. FAAH levels were measured in whole-brain regions of interest using positron emission tomography (PET) with the FAAH-specific probe [^11^C]CURB. An irreversible two-tissue compartment model determined [^11^C]CURB binding. Participants with CUD were scanned once after overnight abstinence (T1) and ~3–7 days after monitored last use (T2). FAAH polymorphism (rs324420) was determined from blood samples, and mood, cognition, withdrawal symptoms, and craving were assessed. In a sample of 14 participants (*N* = 17 prior to attrition) who completed both scans, FAAH binding in whole-brain increased between T1 and T2 (*n* = 14; %ΔFAAH = 10%; *p* = 0.003), with the largest change in the ventral striatum (11%, *p* = 0.026). Increases in FAAH (%ΔFAAH whole-brain) were significantly associated with longer cannabis abstinence, greater baseline depression severity, and tendency to act without thinking (*p* < 0.001). Short-term cannabis abstinence is associated with increases in brain FAAH levels. These changes are linked to traits and symptoms associated with relapse vulnerability, including negative mood and impulsivity. These preliminary findings suggest that FAAH may play a key role in the neurobiological response to short-term abstinence and could represent a potential target for interventions.

## Background

Cannabis is the third most commonly used substance worldwide (after alcohol and tobacco) [1], and its use is particularly common among adolescents and young adults in countries like the United States and Canada [2]. Emerging evidence indicates that the legalization of recreational cannabis is associated with increased use among adults and, in some jurisdictions, modest increases in reported adolescent use [3–5]. This widespread use raises public health concerns [6, 7], yet the effects of cannabis, both beneficial and adverse, are not well understood.

Cannabis use has been linked to physical and mental health issues, as well as societal and economic harm [8, 9]. Approximately one-third of cannabis users experience difficulties related to their use, with about 1 in 10 developing a cannabis use disorder (CUD); this risk rises to 17% for those who first use during adolescence [1, 5, 10]. Cannabis use can also produce withdrawal symptoms—including irritability, anxiety, disrupted sleep, appetite changes, and general discomfort—which can make it difficult to decrease or stop cannabis use [1, 11]. The time course of cannabis withdrawal has been well characterized in clinical studies. Core symptoms—such as irritability, anxiety, low mood, and sleep disturbance—typically begin within 24–72 hours after cessation, increase in severity over the first several days, and peak within approximately 2–6 days of abstinence. While many symptoms gradually diminish after the first week, some, particularly sleep disruption and mood-related symptoms, can persist for up to two weeks or longer in some individuals [11–13]. In parallel, CUD is a leading reason for drug treatment referrals in both the United States [14] and Canada [15]; despite this, current treatment options are often limited to psychosocial interventions [8, 16]. Currently, no pharmaceutical is approved for CUD or cannabis withdrawal treatment, and the neurobiology of withdrawal remains poorly understood, highlighting the need for better understanding of mechanisms and better treatments [1, 17].

Cannabis, containing Δ‑9‑tetrahydrocannabinol (THC), acts mainly on CB1 and CB2 receptors of the endocannabinoid system (ECS). In this system, endogenous ligands such as anandamide (AEA) and 2-arachidonoylglycerol (2‑AG) are synthesized on demand, released from the postsynaptic neuron, and travel retrogradely across the synapse to modulate neurotransmission. These ligands are then degraded by specific enzymes—AEA by fatty acid amide hydrolase (FAAH) and 2‑AG predominantly by monoacylglycerol lipase (MAGL)—thereby tightly regulating ECS signaling and maintaining homeostasis [1, 18].

Chronic cannabis use is robustly associated with CB1 receptor desensitization/downregulation in both preclinical models [19] and human post-mortem as well as in imaging studies [20–22]. For example, positron emission tomography (PET) studies in individuals with CUD show a global reduction in CB1 radiotracers binding (i.e., [^18^F]MK-9470 and [^11^C]OMAR) during the first week of cannabis abstinence. These reductions have been shown to rapidly increase during short-term abstinence ( ~ 48 h) and recovered to normal levels after several weeks [21, 22]. This downregulation of CB1 receptors is thought to contribute to cannabis tolerance and the severity of withdrawal symptoms in CUD [23]. However, the relationship between CB1 receptor regulation and FAAH activity and protein level remains largely unclear. Emerging evidence from cultured neuronal systems suggests that endocannabinoid metabolic pathways, including changes in FAAH protein expression (measured by Western blot), may adapt in concert with CB1 receptor regulation, indicating coordinated homeostatic adjustments within the ECS [24].

Studies investigating the role of FAAH in CUD and cannabis withdrawal suggest a bidirectional (and seemingly contradictory) relationship between FAAH enzyme activity and cannabis-related behaviors. Available evidence suggests that reduced FAAH protein expression and enzyme activity may increase motivation or approach behavior toward cannabis [25, 26]. Human genetic studies indicate that the FAAH missense variant rs324420 (C385A)—which causes a Pro129Thr substitution, reduces FAAH protein stability, leading to lower FAAH protein expression and enzymatic activity, and consequently elevated levels of the endocannabinoid AEA [27, 28] - has been associated with higher rates of cannabis use and CUD [29]. Conversely, enhanced ECS tone produced by FAAH inhibition may help alleviate withdrawal symptoms by compensating for downregulated CB1 receptor signaling [30]. Preclinical studies indeed show that FAAH inhibitors reduce somatic withdrawal signs, dampen anxiety- and stress-related responses, and improve cognitive impairments in animal models of chronic cannabinoid exposure [31, 32]. Consistent with these preclinical findings, FAAH inhibitors recently tested in CUD have been examined for their potential to mitigate withdrawal symptoms [30]. Early findings suggest potential benefits for withdrawal-related symptoms; however, their therapeutic utility in larger clinical trials remains to be established.

Research on the effects of chronic THC exposure and withdrawal on FAAH activity, protein expression, and endocannabinoid tone remain limited. Our imaging studies of FAAH levels (i.e., radiotracer binding) have shown that acute cannabis cessation in CUD is associated with increased plasma AEA [33] and reduced FAAH levels in the brain [29, 34]. The current study aimed to investigate changes in FAAH levels during short-term abstinence and their relationship with withdrawal symptoms. Using positron emission tomography (PET) imaging with the FAAH-specific radioligand [^11^C]CURB [35], we measured FAAH levels in individuals with CUD after overnight abstinence (T1) and again after 3–7 days of monitored abstinence (T2). This tracer developed at our center, demonstrates good specificity for FAAH and excellent test-retest reliability [36].

We hypothesized that FAAH levels, as indicated by [^11^C]CURB binding, would increase in the whole-brain between overnight ( ~ 12 h) cannabis abstinence (T1) and ~3–7 days after last use (T2) in CUD participants. Exploratory analyses examined whether changes in FAAH (%Δ FAAH) correlated with withdrawal symptoms, craving, and cognitive function.

## Methods

### Participant screening

All procedures were approved by the Research Ethics Board (REB #029-2017) at the Center for Addiction and Mental Health (CAMH). Participants were recruited through community flyers, online advertisements, and ongoing studies. Written informed consent was obtained from all participants prior to enrollment.

Current, near-daily cannabis users of all genders aged 16 years and older were eligible for inclusion. Cannabis users were required to meet Diagnostic and Statistical Manual of Mental Disorders (DSM)-5 [37] criteria for current CUD based on Structured Clinical Interview for DSM-5 [38] or score ≥12 on the Cannabis Use Disorders Identification Test (CUDIT-R [39]). The CUDIT-R is a validated tool for assessing problematic cannabis use and probable CUD; a cut-off of 12 or above has been identified as the optimal threshold for diagnosing CUD, aligning with DSM criteria [39]. Recent cannabis use frequency and daily use patterns were assessed with the Daily Sessions, Frequency, Age of Onset, and Quantity of Cannabis Use Inventory (DFAQ-CU) [40]. Mood and anxiety were assessed at screening using the Beck Depression Inventory (BDI) [41], the Patient Health Questionnaire-9 (PHQ-9) for depressive symptoms [42], and the Generalized Anxiety Disorder-7 (GAD-7) [43] and State-Trait Anxiety Inventory (STAI) [44] for anxiety. Impulsivity was assessed using the Barratt Impulsiveness Scale (BIS-11) [45], which measures attentional, motor, and non-planning impulsiveness, and the UPPS-P Impulsive Behavior Scale, which assesses five dimensions of impulsivity: negative urgency, positive urgency, lack of premeditation (tendency to act without thinking), lack of perseverance, and sensation seeking [46].

Exclusion criteria included significant medical conditions, neurological illnesses, history of head trauma, current or past DSM-5 psychotic symptoms, bipolar disorder, severe mood or anxiety disorders, current post-traumatic stress disorder (PTSD), or any substance use disorder other than CUD. Urine samples were collected during screening to rule out current (last 30-days) use of psychoactive drugs (other than cannabis for participants with CUD) and pregnancy. Nicotine/tobacco use was permitted.

### Participant assessments

Participants completed a battery of questionnaires to evaluate craving, withdrawal, and mood and anxiety symptoms during overnight ( ~ 12 hours) abstinence at the first PET scan (T1) and again ~3–7 days after last use, at the second PET scan (T2). These included the Marijuana Withdrawal Checklist (MWC) [13], the Marijuana Craving Questionnaire-Short Form (MCQ-SF) [47], the Profile of Mood States (POMS [48]), PHQ-9 [42], GAD-7 [43] and STAI [44]. A battery of neurocognitive testing was also completed at the two time points to capture measures of cognitive domains that are impacted in individuals with CUD [49, 50], such as processing speed and executive function using (Trail Making Test (A and B) [51]), verbal memory and learning (Hopkins Verbal Learning Test [52]), response inhibition (Stop-Signal Task [53]), working memory (The Digit Span Task (WAIS) [54]), decision-making (Iowa Gambling [55] and Delay Discounting Tasks [56]).

### PET/MRI imaging protocol and data processing

After inclusion, participants completed PET scans with [^11^C]CURB [35]. Cannabis users were required to abstain from tobacco and nicotine vaping overnight ( ~ 12 h) prior to each scan visit (T1and T2) and to refrain from consuming caffeinated beverages on the mornings of the scans to avoid potential confounding effects. On scan days, urine toxicology (BTNX Inc. Rapid Response® 10 Parameter Multi-Drug) was conducted to rule out the use of medications and illicit drugs (excluding cannabis for participants with CUD), and female participants underwent a urine pregnancy test (Rapid Response^TM^). Expired carbon monoxide levels ( < 10 ppm) were measured to confirm the absence of recent smoking [57]; participants with evidence of recent smoking were excluded. The first scan (T1) occurred after overnight cannabis abstinence ( ~ 12 hours), and the second scan (T2) followed ~3–7 days of abstinence. This interval was selected based on evidence indicating that withdrawal symptoms peak within this time frame and to allow flexibility of the PET scan schedule [12].

Radiosynthesis of [^11^C]CURB was performed in-house according to established protocols [35]. PET was conducted on an HRRT brain tomograph (CPS/Siemens, Knoxville, TN, USA). Participants were positioned supine, with their head stabilized using a thermoplastic mask. Following the injection of 360.75 ± 25.9 MBq (9.75 ± 0.7 mCi) of [^11^C]CURB, emission data were collected over a 1-hour period, with sequential frames of increasing duration (scan parameters at T1and T2 are reported in Supplementary Table 1). Images were reconstructed from 2D sinograms using a 2D filtered-back projection algorithm with a HANN filter at the Nyquist cut-off frequency.

Arterial blood radioactivity was measured using an automatic blood sampling system (ABSS; Model PBS-101, Veenstra Instruments, The Netherlands) for the first 22.5 minutes post-injection. Additional arterial blood samples were collected manually at 3, 7, 12, 20-, 30-, 45-, and 60-min post-injection to assess plasma radioactivity and metabolization. A metabolite-corrected plasma curve was used as the input function for kinetic analysis [58]. Blood-to-plasma radioactivity ratios were interpolated using a biexponential function, and the parent plasma fraction was determined using a Hill function.

Each participant also underwent a standard proton density-weighted brain MRI scan using a Discovery MR750 3 T MRI scanner (General Electric, Milwaukee, WI, USA) to delineate and measure volumes of Regions of Interest (ROIs). ROI delineation and kinetic modeling involved extracting time-activity curves from ROIs covering whole-brain using methods outlined in previous studies [58, 59]. ROIs selected for the investigation included the striatum, prefrontal cortex (PFC), hippocampus, anterior cingulate cortex, amygdala, insula, and temporal cortex. The composite parameter for FAAH, λk3 (λk3 = k3 * K1 / k2), was calculated using a two-tissue compartment model with irreversible binding in the second compartment [58]. Whole-brain λk3 was calculated as the weighted mean of all ROIs defined in the template. Changes in FAAH levels (λk3) were calculated using the following formula: %Δ FAAH = ((λk3 T2 − λk3 T1) / λk3 T1) x 100.

### Blood sampling for genotyping and cannabinoid levels

Genotyping for the FAAH polymorphism (rs324420) was performed using venous blood samples collected during PET scans, as FAAH genotype influences the quantification of [^11^C]CURB [60].

THC, related metabolite, and phytocannabinoid quantification were conducted using blood samples collected in gray-topped vacutainers containing an anticoagulant and glycolysis inhibitor (potassium oxalate and sodium fluoride). Samples were collected on both PET scan days. These samples were transferred to polypropylene cryotubes, frozen on dry ice, and stored at –80 °C. Quantification was performed using LC-MS/MS. THC, 11-hydroxy-THC (11-OH-THC), THCCOOH, cannabidiol (CBD), cannabinol (CBN), and cannabigerol (CBG) were quantified as previously described [61, 62].

### Statistical analysis

Data analysis was conducted using the SPSS statistical software package (Version 30.0; IBM Corp., Armonk, NY). Descriptive statistics for the sample that completed the two PET scans, including mean ± standard deviation (or standard error) and range (minimum-maximum), were computed for quantitative variables, while frequencies and percentages were used for categorical variables. Changes in [^11^C]CURB λk3 in ROIs over time (%Δ FAAH; *n* = 14) were evaluated using a repeated measures ANCOVA, with time (T1 and T2) and ROIs as within-subject factors and FAAH genotype as a covariate and expecting a main effect of time. Bonferroni corrections were implemented to adjust for multiple comparisons (of ROI effects) and control the family-wise error rate. Potential confounding factors, such as sex/gender and body mass index (BMI), differences in days of abstinence from cannabis were also considered, and these factors were incorporated into the model only if they were found to significantly affect the outcome. Changes in clinical symptoms, cognitive performance and blood levels of cannabinoids and metabolites were investigated using paired-sample *t* tests to assess the difference between T1 and T2. Multivariable linear regression models were conducted to investigate the relationship between changes in FAAH over time (%Δ FAAH) and clinical characteristics (i.e., drug use patterns, baseline mood, anxiety and trait impulsiveness) and changes in symptoms over time (i.e., mood, craving and withdrawal state). To account for multiple comparisons, false discovery rate (FDR) correction using the Benjamini-Hochberg procedure was applied within each scale family, with a significance threshold of *q* < 0.05.

## Results

Of the 17 individuals with CUD enrolled in the study, 14 participants completed both PET imaging sessions (study completers): T1 following overnight abstinence (17.80 ± 6.2 h) and T2, ~3–7-days following a period of monitored cannabis abstinence (3.96 ± 1.8 days). The mean age at enrollment of study completers (*n* = 14) was 29.9 years (range: 20–45 years), with a majority identifying as male (78.6%). Demographic details for the 14 participants are provided in Table 1.

**Table 1:** Sample demographics.

Demographic variable	*n* = *14*
**Age at enrollment**	29.9 ± 6.5 (20–45)
**Biological Sex at Birth**	
Male	11 (78.6%)
Female	3 (21.4%)
**Body Mass Index (kg/m**^**2**^**)**	27.1 ± 4.4 (20.3–34.3)
**FAAH C385A Genotype**	
AA	2 (14.3%)
AC	9 (64.3%)
CC	3 (21.4%)
**Ethnicity/Race**	
South Asian	1 (7.1%)
White	8 (57.1%)
Hispanic	2 (14.3%)
Multiracial	3 (21.4%)
**Average Years of Education**	15.6 ± 1.9 (12–18)
***Substance Use***	
**Standard Alcohol Drinks Per Week**	9.7 ± 7.5 (1.5–25)
**Lifetime Nicotine/Tobacco**	5 (35.7%)
Cigarette Use (*n*, %; Current/Former)	5 (35.7%; 1/4)
E-cigarette (Vape) Use (*n*, %; Current/Former)	2 (14.3%; 1/1)
**Cannabis Use Characteristics**	
Years Used	12.9 ± 7.2 (4–31)
Age of First Use	16.9 ± 2.3 (14–21)
Current Days of Use Per Week	6.7 ± 0.75 (4.5–7)
Current Dose Per Day (grams)	1.2 ± 1.1 (0.03–3.5)
Current Dose Per Week (grams)	8.3 ± 7.8 (0.01–24.5)
Current Route of Administration	
Joints	12 (85.7%)
Bong / Pipe	5 (35.7%)
Vape	2 (14.3%)
Edibles	2 (14.3%)
**Positive Urine Toxicology**	14 (100.0%)
THC+	14 (100.0%)
Ketamine	1 (7.2%)
**Current/Last 30-Day Medication Use**	5 (35.7%)
Vitamins and/or OTC	4 (28.6%)
SSRI/SNRI	3 (21.4%)
Other^a^	2 (14.3%)
**Last 24mo Medication Use**	6 (42.9%)
Vitamins and/or OTC	1 (7.1%)
SSRI/SNRI	2 (14.3%)
Stimulants	2 (14.3%)
**Current SCID-5 Diagnoses**	
Cannabis Use Disorder (*n*, %; Mild/Moderate/Severe)	14 (100%; 5/5/4)
**SCID-5 Past Lifetime Diagnoses**	
Depressive Disorder	5 (35.7%)
Non-Cannabis Substance Use Disorder	
Alcohol Use Disorder	5 (35.7%)
Stimulant Use Disorder	1 (7.1%)
Hallucinogen Use Disorder	1 (7.1%)
Anxiety Disorder (panic disorder)	1 (7.1%)
Trauma Related Disorders (PTSD)	2 (14.3%)
***Mood, Anxiety, and Affect Questionnaires***	
**Patient Health Questionnaire-9 Screening**	3.4 ± 3.0
**Beck Depression Inventory**^b^	7.2 ± 6.1
**General Anxiety Disorder-7 Screening**	2.9 ± 2.9
**Barratt Impulsivity Scale**^b^	60.6 ± 7.8
**UPPS-P Impulsive Behavior Scale**	
Negative Urgency Subscale^b^	2.0 ± 0.5
(Lack of) Premeditation Subscale^d^	2.1 ± 0.3
(Lack of) Perseverance Subscale^c^	2.0 ± 0.5
Sensation Seeking Subscale^c^	3.0 ± 0.4
Positive Urgency Subscale^c^	1.6 ± 0.4
***Cannabis-Related Questionnaires***	
**Cannabis Use Disorder Identification Test - R Total**	14.5 ± 4.4 (9–22)
**DFAQ-CU Scores**	
Factor 1: Daily Sessions	–0.28 ± 0.39
Factor 2: Frequency	1.37 ± 0.43
Factor 3: Age of Onset^c^	0.70 ± 1.11
Factor 4: Marijuana Quantity	0.23 ± 0.45
Factor 5: Concentrate Quantity	–0.60 ± 0.45
Factor 6: Edible Quantity	–0.21 ± 1.13

*Note*. Continuous variable values presented as Mean ± Standard Deviation. Categorical variables presented as n (%).

Some measures reported in a subset of participants.

^a^Current other medications: *n*Lorazepam = 1, *n*Symbicort = 1, *n*Topimax = 1.

^b^(*n* = 12).

^c^(*n* = 13).

^d^(*n* = 11).

All completers met criteria for current CUD and screened positive for THC on urine dipstick. On average, participants began using cannabis at 16.9 years of age (range: 14–21 years), and used cannabis for an average of 12.9 years (range: 4–31 years). Participants reported using cannabis on average of 6.7 days per week, consuming an average of 1.2 g per day (range: 0.03–3.5 grams). The most common method of cannabis consumption was smoking joints (85.7%). The average CUDIT score was 14.5 (range: 9–22). Participants had no other current psychiatric diagnoses (lifetime psychiatric diagnoses are reported in Table 1).

### Peripheral THC, THC metabolites and minor phytocannabinoids concentrations

Following 3.96 ± 1.8 days (*N* = 14; T2) of self-reported abstinence from cannabis, significant reductions were observed in plasma THC and metabolites (11-OH-THC, THCCOOH) concentrations, corresponding to decreases of –42% (*p* = 0.039), –84% (*p* = 0.015) and –73% (*p* = 0.005) from those at T1. The rate of CBN and CBD detection was low at T1 (*n* = 3; 21% and *n* = 2; 14%) and slightly reduced further at T2 (*n* = 2; 14% and *n* = 2; 14%). In contrast, CBG was detected in more than half the samples at T1 (53%), dropping to 14% at T2 (*p* = 0.12). See Supplementary Table 1.

### Mood, craving, and withdrawal following short abstinence

We conducted repeated-measures ANCOVAs (T1 vs. T2) to assess mood, craving, withdrawal, and cognitive measures, including days of abstinence as a covariate when significant. No significant effects of time were observed for craving factors (MCQ-SF), withdrawal scores (MWC), or cognitive measures. A significant main effect of time was observed for self-reported hostility (POMS subscale: F(1, 12) = 7.495, *p* = 0.018), although pairwise differences between T1 and T2 were not significant. No other significant effects on mood were observed (Supplementary Tables 2–3).

### Changes in brain FAAH levels following short abstinence

A repeated-measures ANCOVA was conducted to examine the main effect of time (T1, T2) on FAAH binding across seven ROIs, controlling for FAAH genotype and days of abstinence. Days of abstinence was entered into the model because it strongly correlated with changes in FAAH levels ([^11^C]CURB λk3) across ROIs, suggesting that longer abstinence is associated with increases in [^11^C]CURB λk3 during cannabis abstinence (*r* = 0.72–0.87; *p* < 0.003). Age, BMI and alcoholic drinks a week did not correlate with FAAH levels ([^11^C]CURB λk3). There was no significant difference in %ΔFAAH between participants with and without a history of mood disorders (all *p* > 0.4).

**Fig. 1:**
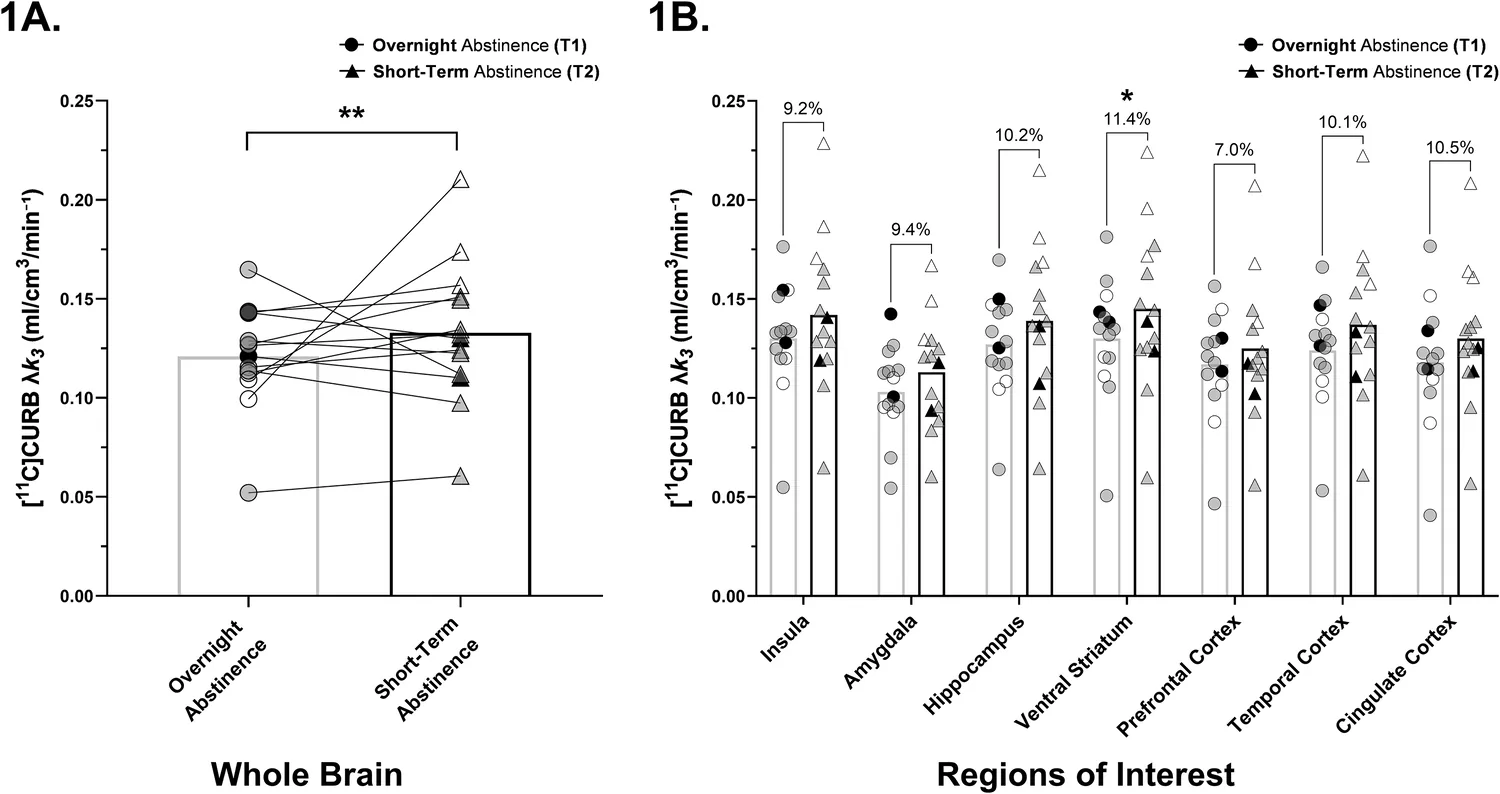
Changes in brain FAAH levels following short-term abstinence. [^11^C]CURB λk3 in whole-brain (**A**) and **B** in 7 regions of interest during overnight (T1; 17.80±6.2 hours) and short-term abstinence (T2; 3.96 ± 1.8 days) in chronic cannabis users (*n* = 14). Mean values are adjusted for days of abstinence and for the FAAH polymorphism. FAAH (rs324420 FAAH C385A) AA: *n* = 2 (Black); AC: *n* = 9 (Shaded); CC: *n* = 3 (Empty) and. Two-tailed significance indicated: ** *p* < 0.005 *p < 0.05.

There was a significant main effect of time (*F*(1, 11) = 14.564, *p* = 0.003; Fig. 1A), a significant time x days of abstinence interaction (*F*(1, 11) = 14.147, *p* = 0.003), and a significant time x ROI x days of abstinence interaction (*F*(6, 66) = 3.910, *p* = 0.002; Fig. 1B), with the greatest change observed in the ventral striatum (11% increase; *p* = 0.026) (Supplementary Fig. 1 for ROI information, which overlays the PET and MRI images to illustrate the anatomical location of the structure).

### Correlates of abstinence-related changes in FAAH binding

A series of multivariate linear regressions were used to investigate the relationship between whole-brain changes in FAAH binding (%ΔFAAH) and withdrawal and clinical measures. The initial model included age, sex, and days of abstinence as covariates; age and sex did not contribute meaningfully and were therefore removed. Days since last cannabis use contributed significantly (*F*(2,13) = 21.656, *p* < 0.001) and was retained for subsequent analyses. To assess whether clinical variables explained additional variance in whole-brain FAAH binding changes beyond days since last cannabis use, we conducted a hierarchical regression model entering days since last use first. Multivariable regression revealed that Depression Severity at baseline (PHQ-9: *R*^2^ = 0.851, *F*(1,11) = 13.722, *p* = 0.003), and Lack of Premeditation (UPPS-P: *R*^2^ = 0.815, *F*(1,8) = 26.174, *p* < 0.001) were significantly associated with changes in whole-brain FAAH (%ΔFAAH) and respectively explained 15.7% and 60% of additional variance beyond days of abstinence. Lack of Self Control (Barratt Impulsiveness Self Control: *R*^2^ = 0.897, *F*(1,8) = 6.412, *p* = 0.035), and changes in emotional craving (MCQ Factor 2: *R*^2^ = 0.797, F(1,11) = 4.313, *p* = 0.062) were marginally associated with changes in whole-brain FAAH and respectively explained 8.3% and 8.0% of additional variance beyond days of abstinence. Changes in FAAH binding during abstinence were not significantly associated with THC analytes, cannabis use severity, mood (POMS), or cognitive test performance (all *p* > 0.05).

## Discussion

In this study, we examined changes in FAAH binding during short-term cannabis abstinence in individuals with CUD and their relationship to mood, withdrawal, cognitive function, and craving. Abstinence led to consistent decreases in THC analytes reaching significance for THC, 11-OH-THC, THCCOOH and subtle increases in self-reported anger but had little effect on overall mood, craving or withdrawal symptoms, likely reflecting low baseline withdrawal severity. The main finding was a significant ( ~ 10%) increase in FAAH binding across brain regions, with the largest change observed in the ventral striatum; exceeding the expected variability based on test–retest measures [36]. Abstinence-related changes in FAAH were not associated with peripheral THC or metabolite concentrations, cannabis use frequency, mood changes, or cognitive performance. However, FAAH increases were related to compulsive and impulsive traits, as well as emotionality-related craving, and were more pronounced in individuals with higher baseline depressive symptoms. Longer durations of abstinence were also associated with greater FAAH increases, suggesting a time-dependent recovery process.

Our findings align with preclinical and clinical evidence that chronic cannabis use leads to adaptations in the ECS. Chronic exposure to Δ9‑THC causes widespread CB1 receptor downregulation and desensitization across multiple brain regions in animals, with somewhat variable regional magnitudes, and human PET studies have shown global decreases in CB1 availability in chronic users that largely reverse with prolonged abstinence, consistent with a global neuroadaptive response to cannabis exposure rather than a strictly ventral striatum‑specific effect [19–22].

Preclinical evidence also demonstrates that chronic THC can alter endocannabinoid concentrations, including increases AEA in limbic forebrain regions that encompass the nucleus accumbens (but not in hippocampus, cerebral cortex or cerebellum), suggesting mesolimbic sensitivity of AEA signaling following cannabinoid exposure [63, 64]. In animals, this region-specific increase may reflect a homeostatic, activity-dependent response by which chronic THC downregulates CB1 receptors, particularly on GABAergic terminals in the ventral striatum, leading to locally enhanced AEA synthesis to restore inhibitory balance and normalize mesolimbic reward signaling.

Some studies also report elevations in AEA and other FAAH substrates (OEA and DHEA) following an overnight abstinence in individuals with CUD [33, 65]. The observed increase in FAAH binding (above test-retest signal [36]) may represent a complementary homeostatic response within the ECS, potentially reflecting enhanced enzymatic capacity to degrade AEA as CB1 signaling begins to normalize. This is consistent with the notion of coordinated regulation between cannabinoid receptors and metabolic enzymes [24].

We speculate that several mechanisms may contribute to the observed FAAH increases. Chronic THC exposure leads first to CB1 receptor desensitization and downregulation [19–22], reducing ECS signaling. In response, AEA levels may initially rise to compensate for lower CB1 activity [33]. During short-term abstinence, as CB1 receptors begin to resensitize [21], FAAH may be upregulated to reduce the transiently elevated AEA, helping to restore synaptic homeostasis and maintain balanced ECS tone. Alternatively, FAAH recovery may be influenced by stress or affective state, as suggested by studies linking FAAH upregulation with increased stress through hypothalamic-pituitary-adrenal (HPA) axis mechanisms [66]. Consistent with this, rodent studies show increases in corticotropin-releasing hormone (CRH) - a key driver of FAAH upregulation [67] - during CB1 antagonist‑elicited cannabis withdrawal [68].

Our study found that individuals with higher baseline depression severity exhibited greater increases in FAAH during short-term cannabis abstinence–a stressful event. This aligns with preclinical evidence showing that depression-like phenotypes and exposure to stress are associated with increased FAAH activity [69], suggesting that FAAH changes during abstinence may be particularly pronounced in individuals with vulnerability to relapse [70].

In the current study, exploratory analyses suggested that participants with higher negative mood also reported greater emotionality-related craving, a known driver of relapse (exploratory analysis not reported in results: *r* = 0.624; *p* = 0.017), and FAAH increases were marginally associated with increases in emotionality-related craving. Together, these findings form a testable model in which FAAH upregulation during short-term abstinence may contribute to relapse risk via heightened emotional craving in individuals with more negative baseline mood, and suggest that individual differences in FAAH regulation could help refine and personalize clinical trials targeting FAAH in CUD. Consistent with this framework, a randomized clinical trial of the FAAH inhibitor PF-04457845 demonstrated reductions in cannabis withdrawal symptoms and cannabis use in a small sample of men, providing early support for FAAH inhibition as a potentially safe and effective therapeutic approach that warrants evaluation in larger trials [30].

Changes in FAAH were however not directly associated with changes in mood, anxiety, or overall withdrawal severity over this short abstinence period. This likely reflects the fact that withdrawal symptoms had not yet peaked; participants reported minimal changes in mood or withdrawal at the second scan. Moreover, the interaction with days since last cannabis use suggests that FAAH-related effects on mood or withdrawal may emerge later in abstinence. Thus, FAAH appears more closely linked to trait-like vulnerability factors such as baseline depressive symptoms and craving rather than momentary state changes in mood or withdrawal.

Our study suggests that increases in whole-brain FAAH binding during cannabis abstinence, are related to higher compulsivity, impulsivity, and emotionality-related craving. These results appear to contrast with prior evidence linking lower (baseline) FAAH activity to greater compulsive drug use, observed in both human genetic studies (e.g., FAAH C385A polymorphism [27]) and animal models [71–73]. This discrepancy can be explained by distinguishing trait-level baseline FAAH from state-dependent FAAH changes during abstinence. Low baseline FAAH may predispose to greater drug use via elevated AEA and enhanced CB1-mediated reward, whereas FAAH upregulation during short-term abstinence in more impulsive or compulsive individuals likely reflects a compensatory response, reducing AEA and normalizing ECS signaling as CB1 receptors resensitize.

The relationship between abstinence duration and FAAH increases suggests a time-dependent plasticity within the ECS. Individuals with longer abstinence intervals showed greater FAAH increases, suggesting that enzyme recovery may continue over several days, although this requires direct testing. Associations with compulsivity, emotionality-related craving, and lower baseline mood indicate that FAAH recovery may be more rapid or pronounced in these individuals, potentially contributing to relapse risk, but this hypothesis remains to be tested.

Key limitations of this study include the small sample size, which may limit statistical power and generalizability, and the short and variable abstinence period, which precludes conclusions about longer-term FAAH recovery. Additionally, and importantly the lack of a control group limits the ability to determine whether observed FAAH levels reflect normal baseline values.

Short-term abstinence from cannabis may be associated with increases in brain FAAH binding, potentially influenced by abstinence duration, compulsivity, impulsivity, and baseline mood. These preliminary findings extend our understanding of ECS plasticity in CUD, suggesting that FAAH could be a dynamic target during early recovery and that individual differences in behavior and affect may modulate enzyme recovery. Future studies with larger samples and longer follow-up are needed to clarify the functional significance of these changes and their implications for treatment development. Complementary designs incorporating imaging during active cannabis use may further elucidate state-dependent FAAH regulation across use and withdrawal.

## Supplementary information

Supplementary Material

## Data Availability

The datasets generated and/or analyzed during the current study are available from the corresponding author on reasonable request.
