# Supplementary material for: PET imaging of FAAH in chronic Cannabis users: longitudinal assessment during short-term abstinence
Source: Neuropsychopharmacology. 2026 May 9;51(11):2124–31. doi: 10.1038/s41386-026-02438-7 (PMC13598130; doi:10.1038/s41386-026-02438-7)
Supplement: Supplementary file 1 [file 41386_2026_2438_MOESM1_ESM.docx]

# **SUPPLEMENTARY MATERIALS**

## **Supplementary Table 1.** Peripheral THC and THC Analyte Concentrations Following Overnight (T1; 17.80±6.2 hours) and Short-Term Cannabis Abstinence (T2; 3.96±1.8 days) in Chronic Cannabis Users (*n*= 14)

| **Compound** | **Overnight Abstinence (T1)** | **Short-Term Abstinence**  **(T2)** | **% Change** | ***p*** |
| --- | --- | --- | --- | --- |
| **THC** | 3.4±3.3 | 2.0±2.4 | -42.1% | **0.039** |
| **11-OH-THC** | 1.3±2.0 | 0.2±0.8 | -83.8% | **0.015** |
| **THCCOOH** | 50.1±53.1 | 13.7±19.9 | -72.6% | **0.005** |
| **Cannabidol** | 0.05±0.1 | 0.01±0.03 | -80.0% | 0.293 |
| **Cannabinol** | 0.04±0.09 | 0.02±0.06 | -44.7% | **0.035** |
| **Cannabigerol** | 0.1±0.1 | 0.03±0.07 | -74.5% | 0.316 |
| *Note.* Values presented as Mean ± Standard Deviation. Paired-sample t-tests (two-tailed) were conducted; statistically significant values are indicated in bold; *p* < .05. Δ9‑Tetrahydrocannabinol (THC), 11‑Hydroxy‑THC (11‑OH‑THC), 11‑Nor‑9‑Carboxy‑THC (THCCOOH), Cannabidiol (CBD), Cannabinol (CBN), Cannabigerol (CBG) | | | | |

## **Supplementary Table 2.** PET Parameters and Clinical Changes Following Overnight (T1; 17.8±6.2 hours) and Short-Term Cannabis Abstinence (T2; 3.96±1.8 days) in Chronic Cannabis Users (*n*= 14)

|  | **Overnight  Abstinence**  **(T1)** | **Short-Term  Abstinence**  **(T2)** | **%  Change** | ***p*** | |
| --- | --- | --- | --- | --- | --- |
| **Duration of Cannabis Abstinence** | 17.80±6.23  (13.6-36.2) hours | 3.96±1.77  (2.5-9.6) days |  | **<.001** | |
| **Scan Parameters** |  |  |  |  | |
| Amount Injected(mCi) | 9.65±0.63 | 9.89±0.65 |  | 0.140 | |
| Ratio of Activity Injected (mCi/kg) | 0.12±0.02 | 0.12±0.02 |  | 0.153 | |
| Specific Activity (mCi/umol) | 1703.93±937.98 | 1574.71±1028.29 |  | 0.505 | |
| Mass Injected (ug) | 2.47±1.62 | 3.66±3.54 |  | 0.167 | |
| ***Mood, Anxiety, and Affect Questionnaires* ^A^** | | | | | |
| **Patient Health Questionnaire-9** | 4.86±1.07 | 4.64±0.87 | -4.4% | 0.779 | |
| **General Anxiety Disorder-7** | 3.93±1.17 | 2.83±0.78 | -27.3% | 0.168 | |
| **State-Trait Anxiety Inventory - State** | 32.07±3.11 | 33.86±2.58 | 5.6% | 0.535 | |
| **Profile of Mood States** |  |  |  |  | |
| Factor 1: Composed – Anxious | 27.86±1.49 | 27.14±1.60 | -2.6% | 0.621 | |
| Factor 2: Elated – Depressed | 25.79±1.64 | 22.71±1.29 | -11.9% | 0.056 | |
| Factor 3: Energetic – Tired | 18.50±2.01 | 19.79±1.74 | 7.0% | 0.511 | |
| Factor 4: Agreeable – Hostile | 27.64±1.51 | 25.86±1.83 | -6.5% | 0.137 | |
| Factor 5: Confident – Unsure | 24.43±1.87 | 23.21±1.45 | -5.0% | 0.333 | |
| Factor 6: Clearheaded – Confused | 26.21±2.18 | 27.79±1.48 | 6.0% | | 0.300 |
| ***Cannabis-Related Questionnaires* ^A^** | | | | | |
| **Marijuana Craving Questionnaire** |  |  |  | |  |
| Factor 1: Compulsivity Sum | 8.07±1.41 | 8.57±1.49 | 6.2% | 0.699 | |
| Factor 2: Emotionality Sum | 9.21±1.63 | 10.71±1.45 | 16.3% | 0.311 | |
| Factor 3: Expectancy Sum | 13.57±1.32 | 12.07±1.39 | -11.1% | 0.111 | |
| Factor 4: Purposefulness Sum | 12.93±1.61 | 13.64±1.49 | 5.5% | 0.574 | |
| **Marijuana Withdrawal Questionnaire** |  |  |  |  | |
| Checklist Total | 9.86±1.72 | 10.93±2.51 | 10.9% | 0.627 | |
| Discomfort Score | 7.64±1.33 | 8.43±1.89 | 10.3% | 0.660 | |
| *Note.* Continuous variable values presented as Mean ± Standard deviation and p values based on paired t-test, unless stated otherwise.  *^A^*Values presented as Mean ± Standard error; Pairwise comparisons were performed on least-squares means adjusted for *Days of Abstinence*, derived from ANCOVA models (significance p < .05). | | | | | |

## **Supplementary Table 3.** Neurocognitive Assessment Following Overnight (T1; 17.8±6.2 hours) and Short-Term Cannabis Abstinence (T2; 3.96±1.8 days) in Chronic Cannabis Users (*n*= 14)

|  | **Overnight  Abstinence**  **(T1)** | **Short-Term  Abstinence**  **(T2)** | **% Change** | ***p*** |
| --- | --- | --- | --- | --- |
| **Trails Making Task** |  |  |  |  |
| Trails A^1^ | 21.62±1.91 | 21.23±1.50 | -6.0% | 0.400 |
| Trails B^2^ | 55.48±5.45 | 57.30±3.92 | 3.3% | 0.636 |
| **Hopkins Verbal Learning Task** |  |  |  |  |
| Total^1^ | 24.86±1.44 | 24.93±1.06 | 0.3% | 0.947 |
| Learning Measure | 4.07±0.43 | 3.43.074 | -15.8% | 0.123 |
| Retained^2^ | 119.20±13.50 | 115.68±7.45 | -3.0% | 0.805 |
| Retained 2^2^ | 9.64±6.74 | 90.36±4.96 | -0.3% | 0.969 |
| Discrimination Index^3^ | 10.00±1.11 | 10.50±0.45 | 5.0% | 0.987 |
| Base Rate^3^ | 0.43±0.05 | 0.53±0.05 | 23.0% | 0.073 |
| Higher of 2 or 3 | 10.00±0.53 | 9.86±0.41 | -1.4% | 0.762 |
| Delay Correct^2^ | 9.00±0.86 | 8.73±0.67 | -3.0% | 0.746 |
| Cued^3^ | 10.60±0.91 | 11.40±0.23 | 7.5% | 0.379 |
| Missed^3^ | 1.40±0.91 | 0.60±0.23 | -57.1% | 0.379 |
| False Positive^3^ | 0.60±0.28 | 0.90±0.28 | 50.0% | 0.472 |
| **Digit Span Task** |  |  |  |  |
| Digit Forward | 11.79±0.58 | 11.50±0.58 | -2.4% | 0.372 |
| Digit Backward | 7.07±0.41 | 7.50±0.63 | 6.1% | 0.401 |
| **Delay Discounting Task - Geomean K** | 0.027±0.008 | 0.025±0.008 | -7.4% | 0.554 |
| **Stop Signal Task**^2^ |  |  |  |  |
| Mean CRT | 666.12±42.34 | 641.46±47.39 | -3.7% | 0.397 |
| Mean PCR | 98.10±0.69 | 96.41±1.13 | -1.7% | 0.111 |
| Mean Delay | 417.84±33.79 | 391.86±61.71 | -6.2% | 0.634 |
| Mean PSI | 55.68±2.05 | 57.82±2.84 | 3.8% | 0.523 |
| Mean SSRT | 248.27±19.55 | 249.59±29.39 | 0.5% | 0.972 |
| **Purdue Pegboard Task - Both Hands** | 12.50±0.74 | 12.29±0.27 | -1.7% | 0.780 |
| **Iowa Gambling Task - Total Earnings**^2^ | -1120.91±400.71 | -307.27±350.07 | -72.6% | 0.091 |
| Values presented as Mean ± Standard error; Pairwise comparisons were performed on least-squares means adjusted for *Days of Abstinence*, derived from ANCOVA models (significance p < .05). Some tests were reported in a subset of participants ^1^(*n*= 13); ^2^(*n*= 11); ^3^(*n*= 10). | | | | |

**Supplementary Figure 1.** Region of Interest (ROI) Delineation and PET Signal in the Ventral Striatum. MRI (**A**) and PET (**B**) images demonstrating ROI delineation of the ventral striatum. Shown in a sample participant (38M, rs324420 FAAH C385A AC, mild CUD severity). Segmented using the ROMI [^11^C]CURB ROI template/brain atlas [1].

**REFERENCES:**

1	Rusjan P, Mamo D, Ginovart N, Hussey D, Vitcu I, Yasuno F, et al. An automated method for the extraction of regional data from PET images. Psychiatry Res. 2006;147(1):79-89.
